# Supplemental dataset on the influence of cardiac resynchronisation therapy in pacing-induced cardiomyopathy and concomitant central sleep Apnea

**DOI:** 10.1016/j.dib.2020.106461

**Published:** 2020-10-28

**Authors:** Fabian Barbieri, Agne Adukauskaite, Thomas Senoner, Andrea Rubatscher, Wilfried Schgör, Markus Stühlinger, Bernhard Erich Pfeifer, Axel Bauer, Florian Hintringer, Wolfgang Dichtl

**Affiliations:** a University Clinic of Internal Medicine III, Medical University Innsbruck, Innsbruck, Austria; b Institute of Clinical Epidemiology, Tirol Kliniken, Innsbruck, Austria; c Institute of Medical Informatics, UMIT TIROL, Eduart Wallnöfer Zentrum, Hall in Tirol, Austria

**Keywords:** Pacing-induced cardiomyopathy, Cardiac resynchronisation therapy, Cardiac remodeling, Sleep-disordered breathing, Central sleep apnea

## Abstract

This article contains supplemental data to the publication “Central Sleep Apnea and Pacing-Induced Cardiomyopathy” [1], which was the most recent publication of the “UPGRADE” study. It provides in-depth analysis of the effects of cardiac resynchronisation therapy (CRT) in patients suffering from pacing-induced cardiomyopathy (PICM) on cardiac remodeling as well as functional cardiac parameters in comparison to continuous right ventricular pacing (RVP). Furthermore, it also covers additional data on several sleep parameters, which were not presented in the main article including the index for obstructive sleep apnea (OSA), the index for mixed sleep apnea and the oxygen saturation measurements during polysomnography. Further, Kaplan-Meier curves are presented for major adverse cardiac events (MACE) and overall mortality by severity of sleep apnea. Generally, the “UGRADE” study was a single-center prospective double-blinded randomized controlled trial lasting from 2014 to 2020. The methodology included a cross-over design giving the possibility to detect differences while CRT was activated and while continuous RVP was applied. The presented data should aid clinicians in daily practice as upgrading to CRT is not limited to improvement in cardiac parameters, but also modifies sleep apnea in patients with PICM, a generally sparsely studied entity of heart failure.

## Specifications Table

**Table:** 

Subject	Medicine and Dentistry
Specific subject area	Cardiology and Cardiovascular Medicine
Type of data	Figure
How data were acquired	Data was collected as part of a single-center prospective double-blinded randomized controlled trial. Analysis was conducted by using IBM SPSS version 24 (IBM Corporation, Armonk, NY, USA). Graphics were designed with GraphPad PRISM, version 5 (GraphPad Software, Inc., La Jolla, CA, USA).
Data format	Raw Analyzed
Parameters for data collection	Inclusion criteria were: Heart failure symptoms despite guideline directed medical therapy (New York Heart Failure Association Class II, III or ambulatory IV) Left ventricular ejection fraction (LVEF) below 40% The need of right ventricular pacing above 40% despite optimal device programming Exclusion criteria were: End-stage heart failure (vasopressor dependent or low output syndrome) Glomerular filtration rate below 30 ml/min/1.73 m2 Life expectancy below one year Women with childbearing potential Drug abuse Hyperthyroidism Intolerance of contrast agent
Description of data collection	Clinical data was collected prospectively at each visit of enrolled patients. Outcome parameters (major adverse cardiovascular events (MACE) and survival rate) were assessed during study visits, the hospital information system and phone calls. Entered data was double checked to reduce the possibility of potential errors.
Data source location	Institution: Medical University Innsbruck City/Town/Region: Innsbruck, Tirol Country: Austria
Data accessibility	With the article
Related research article	Barbieri F, Adukauskaite A, Heidbreder A, Brandauer E, Bergmann M, Stefani A, Holzknecht E, Senoner T, Rubatscher A, Schgör W, Stühlinger M, Pfeifer B, Bauer A, Hintringer F, Högl B, Dichtl W. Central Sleep Apnea in Pacing-Induced Cardiomyopathy. Am J Card. 2020 Sep 28:S0002-9149(20)31002-X. doi:10.1016/j.amjcard.2020.09.027.

## Value of the Data

• The additional data provides further insights of the cardiac remodeling process as well as several other sleep parameters including the respiratory status after activation of CRT in patients with PICM compared to continuous right ventricular pacing.

**Fig. 1:**
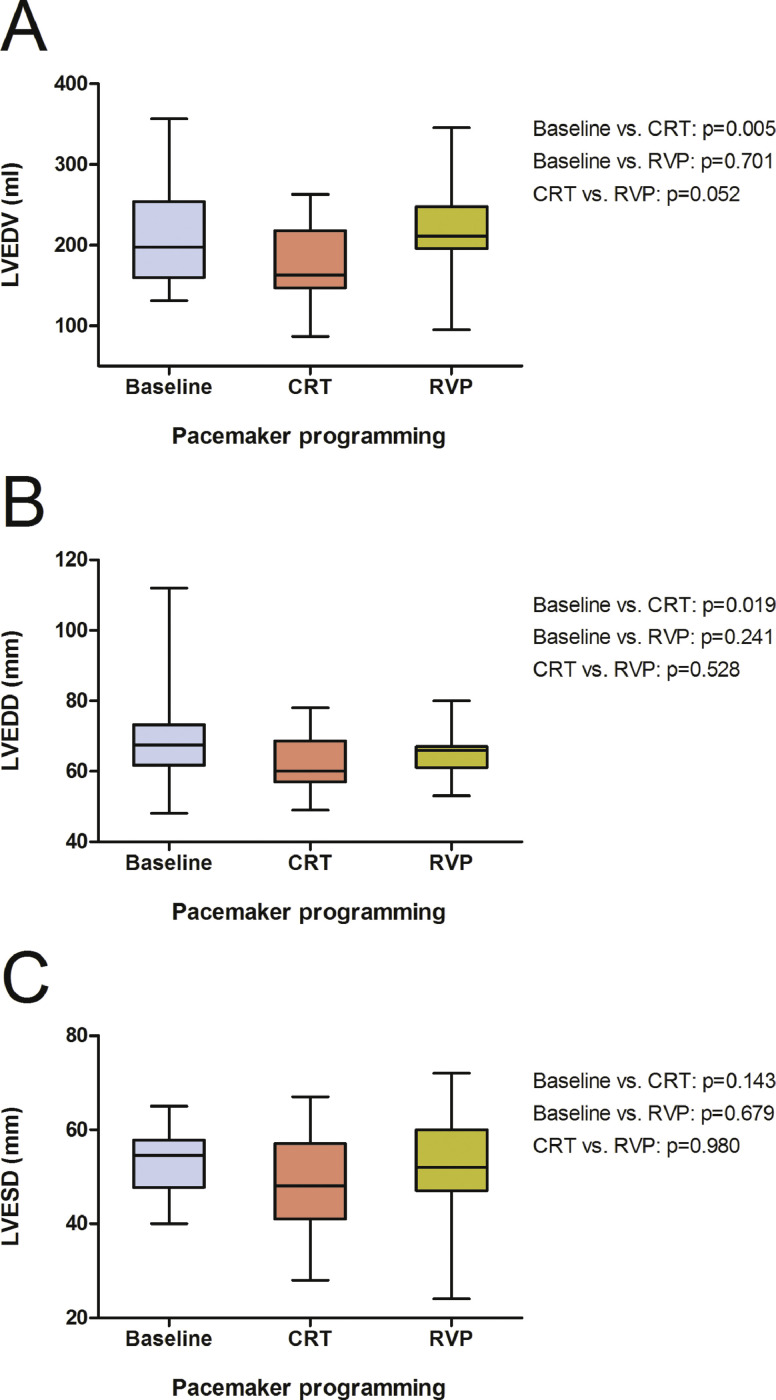
Box plots showing the effects of CRT and continuous RVP on LVEDV (A), LVEDD (B) and LVESD (C). Abbreviations: CRT, cardiac resynchronisation therapy; LVEDD, left ventricular end diastolic diameter; LVEDV, left ventricular end diastolic volume; LVESD, left ventricular end systolic diameter; RVP, right ventricular pacing.
• These results should provide guidance to clinicians working regularly with cardiac pacemakers facing patients with PICM. An amelioration of several cardiac, but also sleep parameters can be achieved by upgrading to CRT.
• The data described should help to understand the complex interplay between heart failure and sleep apnea. It further underlines the open issues to be adressed in future trials, but also helps to estimate the treatment effect for power analysis.

## 1 Data Description

**Fig. 2:**
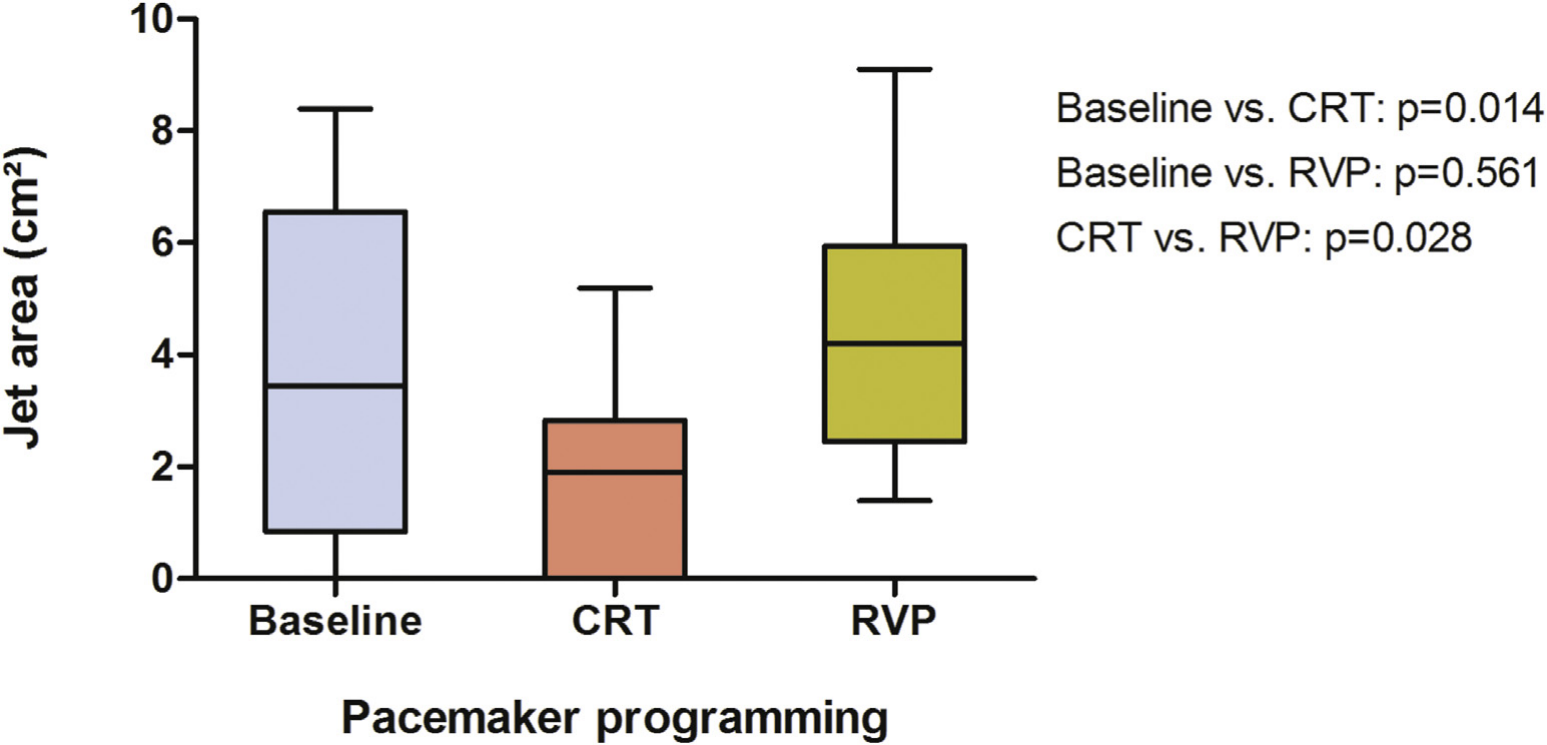
Box plots showing the effects of CRT and continuous RVP on mitral regurgitation jet area. Abbreviations: CRT, cardiac resynchronisation therapy; RVP, right ventricular pacing.

**Fig. 3:**
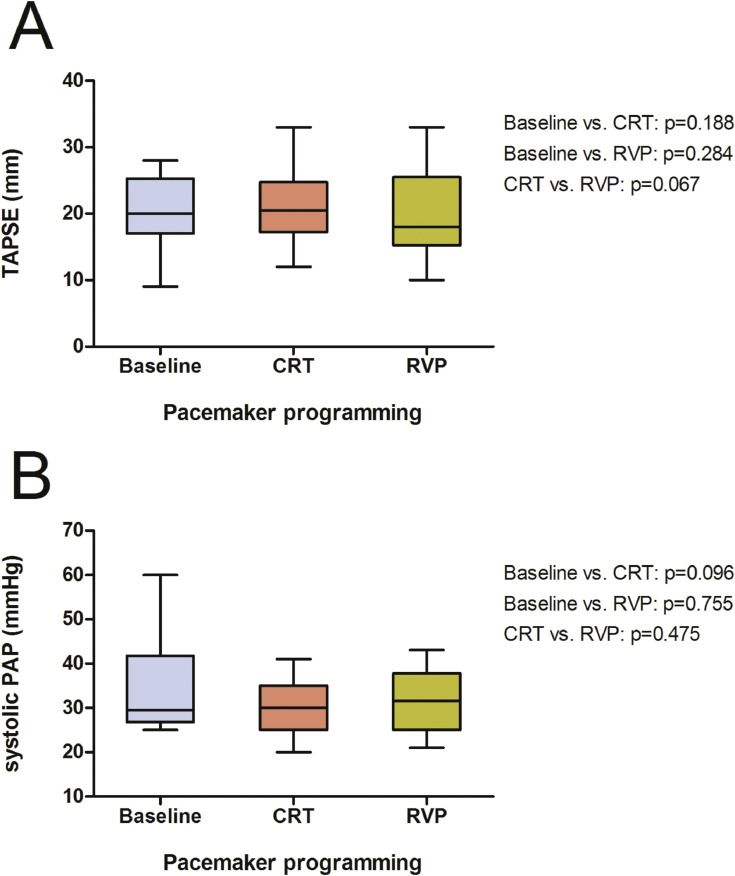
Box plots showing the effects of CRT and continuous RVP on TAPSE (A) and systolic PAP (B). Abbreviations: CRT, cardiac resynchronisation therapy; PAP, pulmonary arterial pressure; RVP, right ventricular pacing; TAPSE, tricuspid annular plane systolic excursion.

Besides the already presented structural improvement in left ventricular ejection fraction (LVEF) and left ventricular end systolic volume [1], there was also a significant reduction in left ventricular end diastolic volume (LVEDV, 198 ml, 160–254; Fig. 1, A) and left ventricular end diastolic diameter (LVEDD, 68 mm, 62–73; Fig. 1, B) after activation of CRT (163 ml, 147–218, *p* = 0.005; 60 mm, 57–69, *p* = 0.019), whereas no effect was yielded under continuous RVP (211 ml, 196–248, *p* = 0.701; 66 mm, 61–67, *p* = 0.241). Although showing similar tendencies, the reduction in left ventricular end systolic diameter (LVESD, 55 mm, 48–58; Fig. 1, C) due to CRT did not reach statistical significance (48 mm, 41–57, *p* = 0.143). RVP did not show any benefit (52 mm, 47–60, *p* = 0.980). Further, secondary mitral regurgitation (jet area 3.9 cm², 0.6–6.8; Fig. 2) was significantly reduced by CRT (1.9 cm², 0–2.8, *p* = 0.014), on the contrary continuous RVP did not yield any improvement (4.2 cm², 2.5–6.0, *p* = 0.561). As shown in Fig. 3, right ventricular function measured by tricuspid annular plane systolic excursion (TAPSE, 20 mm, 17–25) and the calculated systolic pulmonary arterial pressure (30 mmHg, 27–42) were neither influenced by CRT (21 mm, 17–25, *p* = 0.188; 30 mmHg, 25–35, *p* = 0.096) nor by RVP (18 mm, 15–26, *p* = 0.284; 32 mmHg, 25–38, *p* = 0.755).

**Fig. 4:**
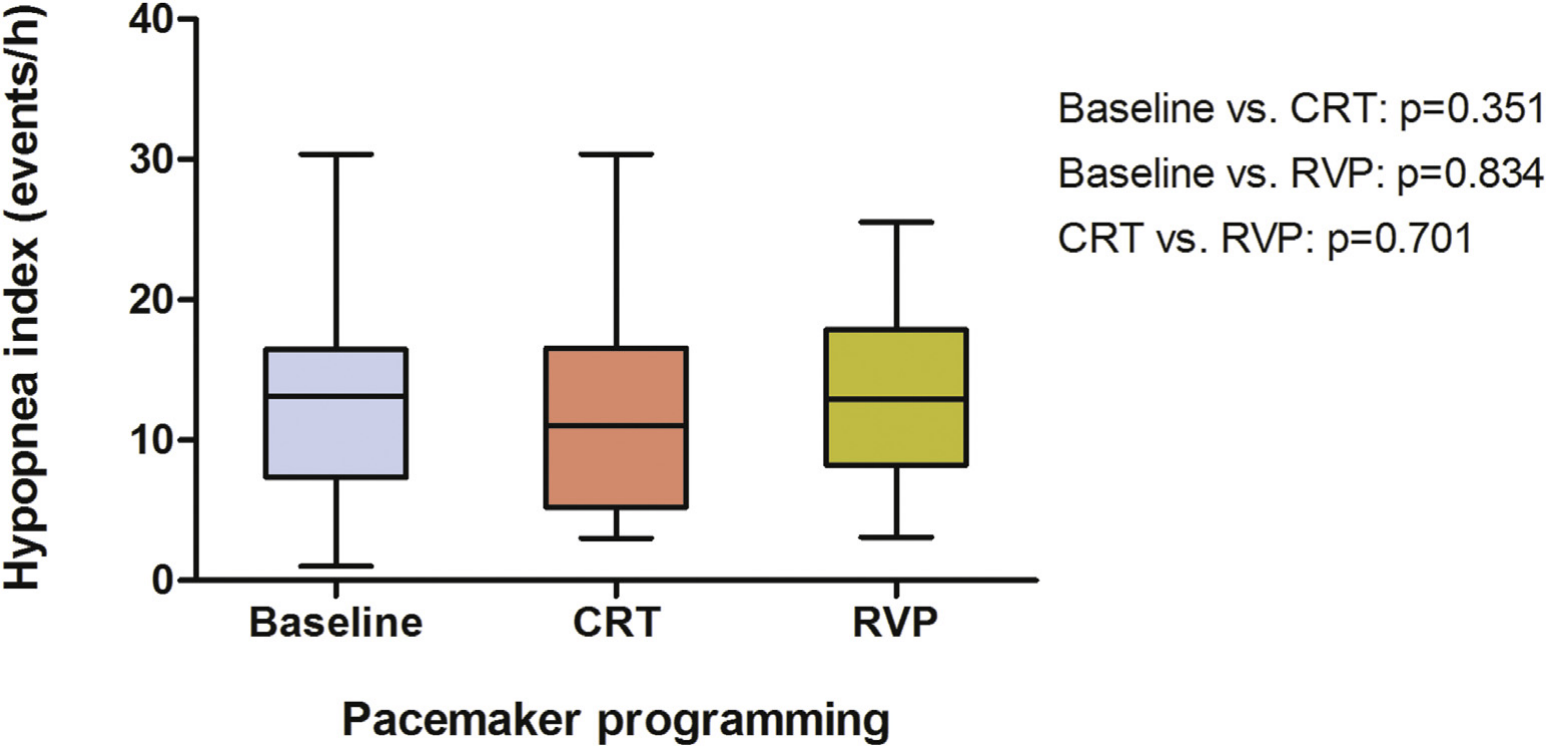
Box plots showing the effects of CRT and continuous RVP on the hypopnea index. Abbreviations: CRT, cardiac resynchronisation therapy; RVP, right ventricular pacing.

**Fig. 5:**
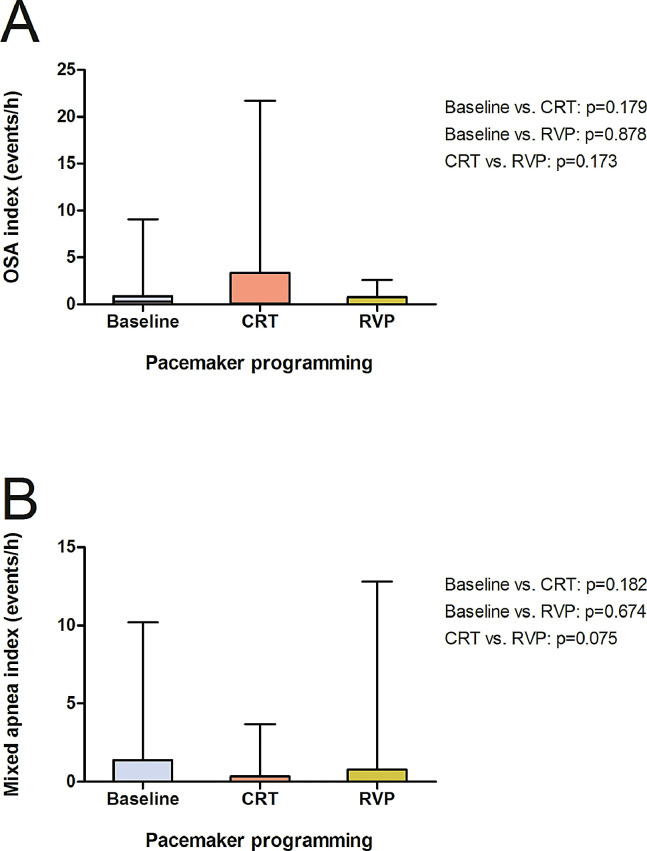
Box plots showing the effects of CRT and continuous RVP on the index for OSA (A) and the index for mixed apnea (B). Abbreviations: CRT, cardiac resynchronisation therapy; OSA, obstructive sleep apnea; RVP, right ventricular pacing.

**Fig. 6:**
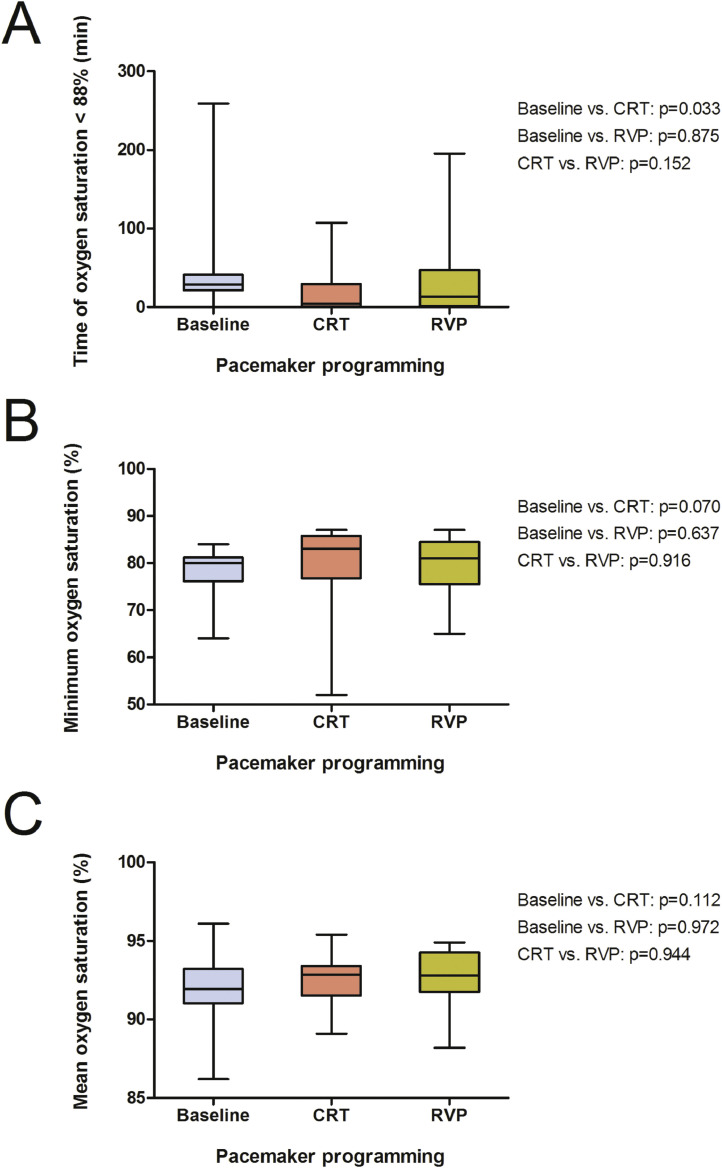
Box plots showing the effects of CRT and continuous RVP on time of oxygen saturation below 88%, minimum oxygen saturation and mean oxygen saturation during polysomnography. Abbreviations: CRT, cardiac resynchronisation therapy; RVP, right ventricular pacing.

**Fig. 7:**
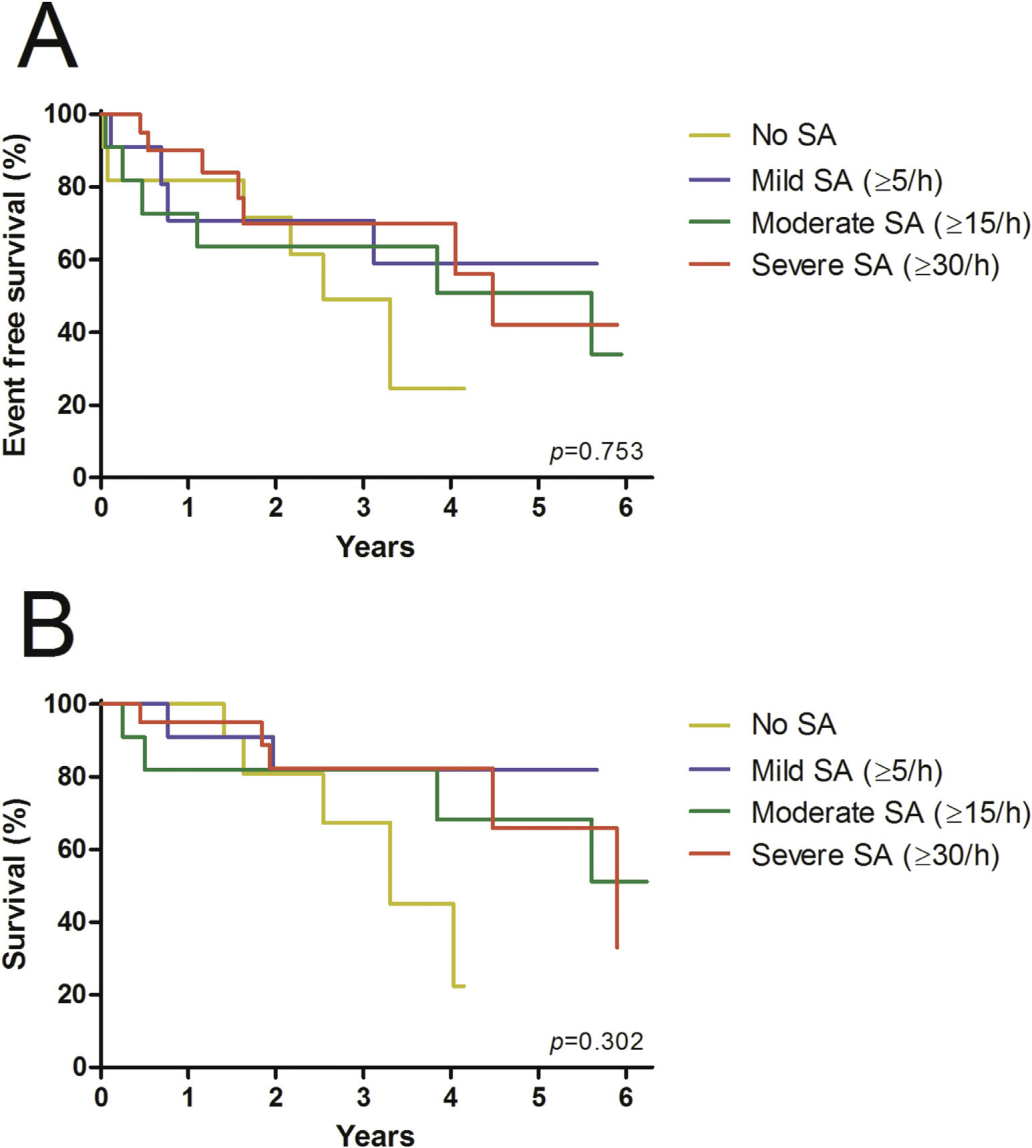
Kaplan-Meier estimates of MACE (A) and overall mortality rates (B), stratified to severity of SA. Abbreviations: MACE, major adverse cardiac events; SA, sleep apnea.

There was not significant influence on the hypopnea index (13.2/h, 7.4–16.5; Fig. 4) by either pacing modality (CRT: 11.0/h, 5.2–16.6, *p* = 0.351; RVP: 12.9/h, 8.3–17.9, *p* = 0.834). Similar results were found regarding the index for OSA (0.3/h, 0–0.8) and mixed sleep apnea (0/h, 0–1.4) as CRT (0.7/h, 0–3.4, *p* = 0.179; 0/h, 0–0.3, *p* = 0.182) and RVP (0/h, 0–0.8, *p* = 0.878; 0/h, 0–0.8, *p* = 0.674) failed to display any differences (Fig. 5, A and B). The time below an oxygen saturation of 88% (28.9 min, 21.3–41.4; Fig. 6, A) during sleep was significantly reduced by CRT (4.2 min, 0.9–29.5, *p* = 0.033), whereas no effect was yielded under RVP (13.1 min, 1.2–46.9, *p* = 0.875). There was also a similiar trend to improvement in minimum oxygen saturation (80.0%, 76.2–81.2; Fig. 6, B) and mean oxygen saturation (92.0%, 91.0–93.2; Fig. 6, C) due to CRT (83.0%, 76.8–85.8, *p* = 0.070; 92.9%, 91.5–93.4, *p* = 0.112), although it did not reach statistical significance. No effect was seen under continuous RVP (81.0%, 75.5–84.5, *p* = 0.637; 92.8%, 91.8–94.3, *p* = 0.972). Severity of sleep apnea had no impact on MACE (*p* = 0.753, Fig. 7, A) or all-cause mortality (*p* = 0.302, Fig. 7, B) during mid-term follow up.

## 2 Experimental Design, Materials and Methods

The UPGRADE study (ClinicalTrials.gov Identifier: NCT01970423) was an investigator-driven single-center prospective double-blinded randomized controlled study. The major purposes of this trial were the validation of the AP scan®, a novel algorithm implemented in cardiac pacemakers to screen for sleep apnea [2], and to evaluate the effects of CRT upgrading in patients suffering from PICM and concomitant CSA. Inclusion criteria were heart failure symptoms despite guideline directed medical therapy (New York Heart Failure Association Class II, III or ambulatory IV), a LVEF below 40% and the need of right ventricular pacing above 40% despite optimal device programming. Exclusion criteria were end-stage heart failure (vasopressor dependent or low output syndrome), a glomerular filtration rate below 30 ml/min/1.73 m2, a life expectancy below one year, women with childbearing potential, drug abuse, hyperthyroidism, and intolerance of contrast agent. The enrolment phase started in 2014 and was completed in 2019, the last patient completed the follow-up in 2020. A detailed description of the conduction of study specific examinations as well as the follow-up schedule is presented in the related research article [1]. Following formulas were used to calculate index for OSA [(number of obstructive apneas) × 60 / total sleep time (in minutes)] and the index for mixed apnea [number of mixed apneas × 60 / total sleep time (in minutes)]. Statistical analysis was conducted using IBM SPSS, version 24 (IBM Corporation, Armonk, NY, USA), graphics were designed using GraphPad PRISM, version 5 (GraphPad Software, Inc., La Jolla, CA, USA). Continuous variables are displayed as median (interquartile range), categorical variables are expressed as number (percentage). Distribution of continuous variables was assessed by inspection of histograms and using the Kolmogorov-Smirnov test. Differences in repeated measurements were analysed either with the paired *t*-test or the Wilcoxon test, according to their distribution. Analysis of MACE and survival were estimated with the Kaplan-Meier method, differences were assessed with the log-rank test. Whiskers in the boxplot demonstrate the 95% percentile (2.5–97.5). *p*-values ≤ 0.05 were regarded statistically significant.

## CRediT Author Statement

**Fabian Barbieri:** Conceptualization, Investigation, Resources, Formal analysis, Writing - original draft. **Agne Adukauskaite:** Writing - original draft, Investigation, Methodology. **Thomas Senoner:** Investigation, Formal analysis, Data curation, Methodology. **Andrea Rubatscher:** Investigation, Data curation. **Wilfried Schgör:** Investigation. **Markus Stühlinger:** Investigation, Writing - review & editing. **Bernhard Erich Pfeifer:** Software, Validation. **Axel Bauer:** Resources, Supervision. **Florian Hintringer:** Conceptualization, Resources, Supervision, Writing - review & editing. **Wolfgang Dichtl:** Conceptualization, Writing - review & editing, Project administration, Funding acquisition

## 3 Ethics Statement

The protocol of this trial was approved by the local ethics committee. The study was conducted in accordance with the “Declaration of Helsinki”, verbal and written informed consent was obtained from each patient enrolled into the study.

## Declaration of Competing Interest

This study has received a grant by the Austrian National Bank “Jubiläumsfondsprojekt Nr. 15,974″. Further it was supported by an unlimited scientific grant from the Boston Scientific Investigator Sponsored Research (ISR) Committee, Boston Scientific, St. Paul, MN, USA. Both grants were assigned to Wolfgang Dichtl.

## Appendix Supplementary materials

Image, application 1
